# Enterobacter Meningitis Due To Dermoid Cyst Manipulation

**Published:** 2018

**Authors:** Sedigheh RAFIEI TABATABEI, Roxana AZMA, Manijeh KAHBAZI, Abdonaser FARZAN, Maryam KAZEMI AGHDAM, Kimia SEIFI, Negin NAHANMOGHADDAM

**Affiliations:** 1 Pediatric Infections Research Center, Research Institute for Children Health, Shahid Beheshti University of Medical Sciences, Tehran, Iran; 2 Department of Radiology, Mofid Children’s Hospital, Shahid Beheshti University of Medical Sciences, Tehran, Iran; 3 Infectious Diseases Research Center (IDRC), Arak University of Medical Sciences, Arak, Iran; 4 Department of Neurosurgery, Shahid Beheshti University of Medical Sciences, Tehran, Iran; 5 Pediatric Pathology Research Center, Mofid Children’s Hospital, Shahid Beheshti University of Medical Sciences, Tehran, Iran; 6 Young Researchers and Elite Club, Roudehen Branch, Islamic Azad University, Roudehen, Iran; 7 Department of Pediatrics, Bouali Children’s Hospital, School of Medicine, Ardabil University of Medical Sciences, Ardabil, Iran

**Keywords:** Meningitis, *Enterobacter*, Antibacterial agents, Dermoid cyst

## Abstract

Gram-negative meningitis can occurssubsequent to dura-arachnoid barrier disruption because of trauma, surgery and rarely an infected dermoid cyst. Association of neurosurgical procedures with Gram-negative meningitis was described for the first time in 1940. Intracranial infections from gram-negative bacilli like *Enterobacter* are serious and difficult to treat as many antibiotics fail to achieve bactericidal concentrations in the cerebrospinal fluid. Here in, we report a rare case of pediatric *Enterobacter *meningitis in a patient with a dermoid cyst that had been manipulated. She was managed with antibiotic therapy plus surgical removal of the infected cyst.

## Introduction

*Enterobacter* is a genus of Enterobacteriaceae that is an increasingly frequent cause of healthcare-associated pediatric infections. It can cause infection of postsurgical wounds, gastrointestinal, urinary and respiratory tracts and meningitis.

*E. cloacae *and* E. aerogenes* are the most common species recovered from clinical specimens. Additional *Enterobacter *species rarely recovered from human infections include *E. amnigenus, E. asburiae, E. gergoviae, E. cancerogenus *and *E. kobei. E. ludwigii* is a new species isolated from clinical specimens closely related to the E. cloacae complex (1).

Gram-negative bacilli may cause meningitis, but it is rare beyond neonatal period and history of impaired immune system or infected brain cyst due to neurosurgical manipulation that lead to CSF contamination are prerequisites to the infection (2, 3).

Risk factors for *Enterobacter* meningitis have not been clearly defined yet (4) but prolonged antibiotic administration and cyst manipulation over the scalp are the two main factors associated with *Enterobacter *meningitis (5, 6). Meningitis due to *Enterobacter* species is an uncommon infection after neonatal period without underlying risk factors so a search for risk factors must be done in all cases of *Enterobacter *meningitis (6).

Intracranial fossa dermoid cysts are congenital benign masses with a prevalence of 0.1%-0.7% of all intracranial neoplasms (7, 8). The presence of dermoid cyst in the posterior fossa is uncommon and surgery is the treatment of choice. Manipulated dermoid cyst should be resected by surgery in addition to antibiotic therapy (8, 9).

## Case Report

An 11-year old girl was admitted Mofid Children**’s** Hospital, Tehran, Iran in 2015 and we took her family informed consent form template for case report studies. She had high-grade fever, severe headache, and vomiting since four days before admission. She had been diagnosed with meningitis in another hospital and had received dexamethasone and antibiotics including ceftriaxone and vancomycin. No improvement had been seen in her status, and her high-grade fever had remained uncontrolled. The patient was referred to Mofid Hospital with complaints of high-grade fever, severe headacheand photophobia. Her mother reported that she had a history of common cold, sinusitis and pharyngitis few days before her first admission.

In addition, she had a history of right knee arthritis last year; she also had a congenital occipital mass with occasional purulent discharge. It had been manipulated by her father because of pain and edema 2 weeks before her admission leading to oozing of pus.

On physical examination, the patient was lethargic; vital signs included body temperature of 39 °C, blood pressure120/80 mm Hg, pulse rate 90/min, and respiratory rate 25/min. Meningeal irritation was present (positive nuchal rigidity, Kernig and Brudzinski signs) with photophobia; pupils midsize and were reactive to light. Erythematous occipital mass (1×2 cm) was palpated in the midline, with no discharge. The range of motion of right hip was decreased with tenderness without erythema, warmth and edema. The rest of the physical examination was normal.

Because of continuation of fever despite antibiotic administration, lumbar puncture was done. CSF analysis in previous hospital admission showed; WBC=132(p=70%,L=28%), Glucose=10, Protein=27, RBC=180 and with a gram-negative bacilli (*Enterobacter)* in CSF culture.

Laboratory evaluations showed WBC=17000/mm^3^ (PMN=87%), Hemoglobin=12.6 g/dl, and Platelets=197000/mm^3^. Biochemistry tests including Na, K, P, amylase, lipase, and LDH were normal and serology for hepatitis b, hepatitis Cand CMV viruses was reported as negative. The erythrocyte sedimentation rate, (ESR) was 67 mm in the first hour and CRP 124 mg/l. NBT, Immune Globulins (Igs) and CD flow cytometry were also in normal range. Mild TR, Mild AIand Mild PI were reported in echocardiography without any vegetation. Audiometry also was normal.

CSF analysis in our hospital showed WBC=1000/mm^3^ with 90% PMN and 10% lymphocyte, Glucose=18mg/dl, Protein=30 mg/dl, RBC=10/mm^3^and CSF culture was negative. 

Antibiotics were changed to vancomycin and meropenem in our hospital. After 3 days, the patient was not responding and had leukocytosis and fever, so chloramphenicol was added to vancomycin and meropenem. Five days later, CSF analysis showed WBC=100/ mm^3^ with 80% PMN and 20% lymphocyte, Glucose=25 mg/dl, Protein=18 mg/dl, RBC=60/mm^3^Blood culture was negative. Vancomycin and meropenem were discontinued and intrathecal amikacin was added to chloramphenicol. After five days, CSF analysis showed WBC=30/mm^3^with40% PMN and 60% lymphocyte, Glucose=29mg/dl, Protein=95mg/dl, RBC=10/ ml. The second CSF culture 8 days after admission showed gramnegative bacilli *Enterobacter* by BACTEC (Becton Dickinson Microbiology Systems, Model No. B9120). According to the antibiogram, microorganism was sensitive to chloramphenicol and cotrimoxazole and resistant to ampicillin- sulbactam, amikacin, gentamicin and cefepime. Therefore, antibiotics were changed based on antibiotic sensitivity test (Amikacin was discontinued, chloramphenicol continued and cotrimoxazole added). Patient responded to the new antibiotic therapy clinically and CSF culture became negative. 

**Figure 1:**
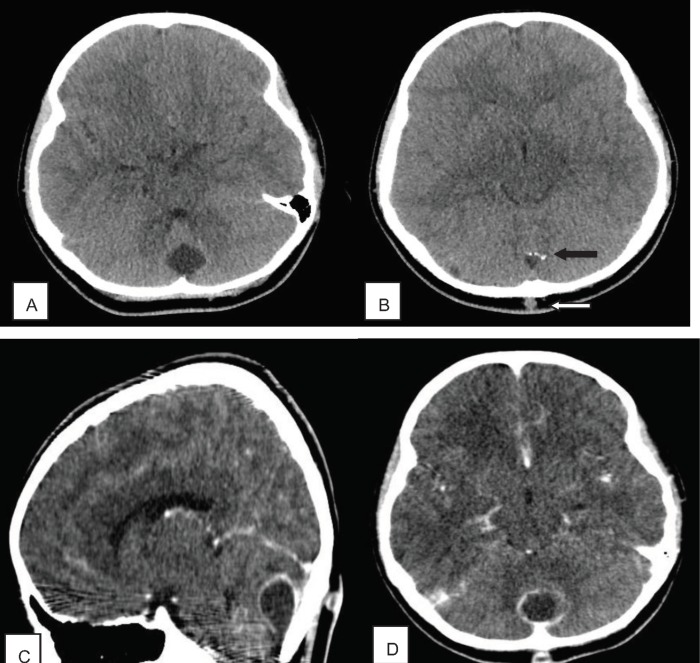
A and B axial non-enhanced brain CT scan show an extra-axial hypodense relatively round lesion in posterior mid aspect of posterior fossa. Note the calcified foci in superior aspect of the lesion(black arrow) as well as a small subcutaneous lesion in the overlying scalp(white arrow)

**Figure 2:**
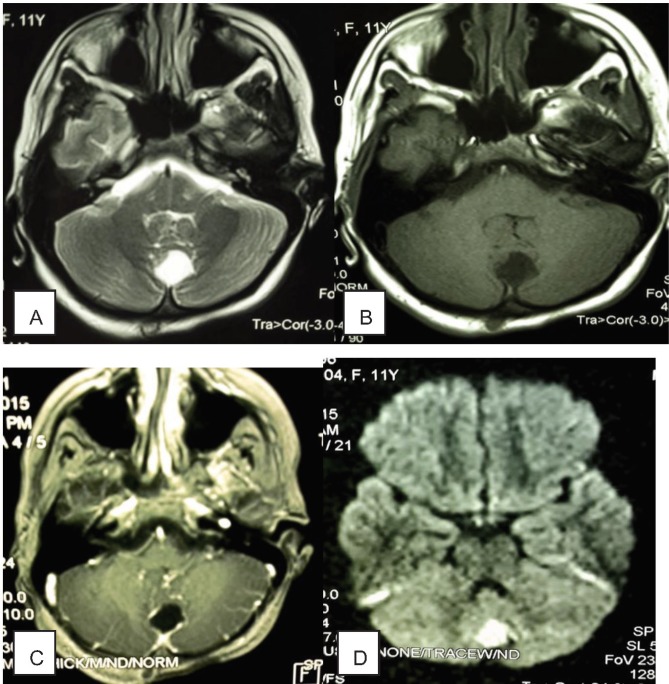
**A. **Axial T2 Weighted brain MRI and B. Axial T1weighted brain MRI shows an extra-axial CSF density lesion in posterior mid aspect of posterior fossa. C. Axial T1 Weighted contrast enhanced brain MRI reveals rim enhancement of the lesion. D. DWI (Diffusion Weighted Imaging)sequence demonstrates restriction in the lesion.

Brain CT scan with and without contrast was performed for the patient which showed an extra-axial hypo dense relatively round lesion in posterior mid aspect of posterior fossa along with calcified foci in its superior aspect. A subcutaneous lesion was also noted in the overlying scalp. After contrast administration, rim enhancement was seen in both intracranial and subcutaneous lesions (Figure 1). The lesion was suspected to be an infected intracranial and subcutaneous dermoid cyst with a connecting tract through the occipital bone, Brain MRI with and without contrast was performed subsequently which revealed CSF signal in the lesion and rim enhancement as well as prominent restriction in DWI sequence (Diffusion weighted imaging) which confirmed the diagnosis in the CT scan (Figure 2).

She was referred to the Neurosurgical Department for removal of the infected dermoid cyst. After neurosurgery, Brain CT revealed an evidence of posterior craniotomy in occipital bones with mild pneumocephalus. Small subdural effusion was detected in the right side of the posterior fossa with normal ventricle sulci; no infarction, hemorrhage, mass, midline shift or herniation was seen.

C. Coronal contrast-enhanced and D. axial contrast-enhanced brain CT scan reveal rim enhancement of both intracranial and subcutaneous lesions.

**Figure 3:**
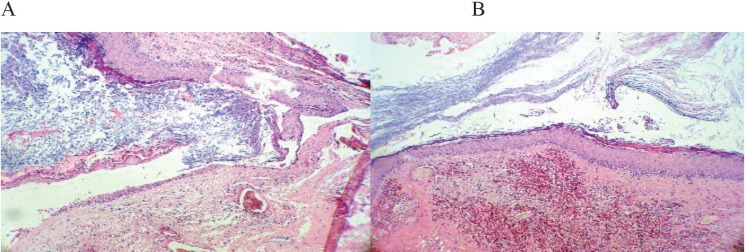
Cyst wall and intracystic keratinous material (A), squamous epithelium of the cyst wall and hair shafts in underlying tissue (B).

The histopathology findings of brain mass showed a cyst lined by keratinized squamous epithelium containing hair shafts and keratinous material. Severe mixed inflammation and foreign body type reactions well as foci of calcification with hyalinization and congestion in the periphery are seen with no evidence of malignancy. It confirms the diagnosis of an infected dermoid cyst (Figure 3).

She was discharged with final laboratory results of normal CBC, CRP=23and ESR=60. Her final CSF analysis showed negative smear and culture. Meningitis was treated by chloramphenicol and cotrimoxazole. Patient responded to our treatment and was well on discharge and on follow-up one month after discharge with normal CBC, ESR and CRP.

## Discussion

Infected idiopathic and congenital brain lesions can cause meningitis, following a defect through the defense barrier of brain(2).As it provides an entry of organisms to invade the meninges (10). Although gram-negative bacilli are the most common cause of nosocomial meningitis, it still remains an unusual cause of meningitis in adults and children (4). Major risk factors include cerebrospinal fluid leak, immunosuppressed patients, history of immunosuppressive drugs consumption, presence of infected brain mass and head trauma or history of neurosurgery (11). Gram-negative bacillary meningitis has mortality rates of 40 to 80% among adults and children (12).*Enterobacter* meningitis dominantly occurs in cases with neurosurgical brain mass manipulation and trauma (13).

Intracranial dermoid cysts are rare, usually located in the posterior fossa and are slow growing (14, 15).These presents with headache and seizure and are usually reported in the first and second decades of life (16). Dermoid cyst of the posterior fossa may be occurring accompanied with Staphylococcus epidermidis abscess. It cured with radical excision of the occipital cyst followed by antibiotics therapy (17).

*Enterobacter* may be found in manipulated mass in the brain. It is a serious infection and resection of the mass and appropriate antibiotic therapy based on culture is the treatment of choice (18). *Enterobacter* was one of the rare and important causes of nosocomial bacteremia in hospitalized children (19). Our patient presented with *Enterobacter* meningitis at age 11 year. Gram-negative bacilli meningitis is a causative agent in the neonatal period but occurrence in adults without immunologic risk factors is very rare (20).

Antimicrobial regimens for treatment of *Enterobacter* meningitis iscontroversial. The efficiency of cefepime in postoperative meningitis attributable to *Enterobacter* has been described (17). In the United States, 15 patients who had positive cultures for *Enterobacter* were evaluated (4). The main treatments of these patients included intravenous (I.V) carbapenems (60% of cases were treated with carbapenems with both I.V. monotherapy and combined I.V- protocols). In addition, one patient was treated with a third-generation cephalosporin, 1 patient with piperacillin and 2 patients with ciprofloxcacin. Patients treated with combined I.V. therapies received the following regimens: (6 patients) were given aminoglycoside in addition to intravenous carbapenem (5 patients) or third-generation cephalosporin (1 patients), and 1 patient was treated by intravenous carbapenem and a third-generation cephalosporin. Among all, one patient had died in I.V combination therapy (4). A 22-year-old patient underwent neurosurgical manipulations developed *Enterobacter *meningitis who was treated with tobramycin, I.V. ceftriaxone, vancomycin, and metronidazole (10). However, in our study, the cultured bacteria were resistant to aminoglycosides and beta-lactam antibiotics that led to the lack of response tointrathecal amikacin and I.V. vancomycin and meropenem. On the other hand, chloramphenicol and cotrimoxazole aresusceptible against gram-negative bacilli and also achieve high concentrations in CSF (21), which the case of which our patient was finally cured with combination of chloramphenicol and co -trimoxazole.

Korinek et al. designed a prospective trial of 3,000 patients reported Staphylococcus aureus was found as a most common cause of meningitis in infected brain masses and the second common pathogen was Enterobacter in 24% cases (22). Enterobacter was isolated from CSF in 16% of patients who enrolled in Parodi’s study (4).

Dermoid cyst of the posterior fossa is described in a 16-yr-old man; who presented with clinical signs of intracranial pressure and cerebellar symptoms (8). They showed there is no risk of infectious complications without manipulation similar to our patient showed the symptoms and signs of meningitis due to dermoid cyst manipulation.

In conclusion, effective antibiotic therapy is crucial for the treatment of *Enterobacter *meningitis. Although the first step is initiating antibiotics but with the presence of an infected mass, surgery is necessary for removal of the mass. Our patient identified with dermoid cyst, which had been infected owing to manipulation. Appropriate antibiotic therapy cured our patient beside neurosurgery removal of cyst to eliminate the underlying factor. 
